# Sulfuretin Attenuates MPP^+^-Induced Neurotoxicity through Akt/GSK3β and ERK Signaling Pathways

**DOI:** 10.3390/ijms18122753

**Published:** 2017-12-19

**Authors:** Ramesh Pariyar, Ramakanta Lamichhane, Hyun Ju Jung, Sung Yeon Kim, Jungwon Seo

**Affiliations:** 1 Institute of Pharmaceutical Research and Development, College of Pharmacy, Wonkwang University, Iksan 570-749, Korea; ume.ramesh@gmail.com (R.P.); sungykim@wku.ac.kr (S.Y.K.); 2 Hanbang Body-Fluid Research Center, Wonkwang University, Iksan 570-749, Korea; 3 Deptartment of Oriental Pharmacy, & Wonkwang-Oriental Medicines Research Institute, College of Pharmacy, Wonkwang University, Iksan 570-749, Korea; clickrama@gmail.com (R.L.); hyun104@wku.ac.kr (H.J.J.)

**Keywords:** sulfuretin, Parkinson’s disease, MPP^+^, apoptosis, Akt, GSK3β, ERK, p53

## Abstract

Parkinson’s disease (PD) is the second most common neurodegenerative disease. It is caused by the death of dopaminergic neurons in the substantia nigra pars compacta. Oxidative stress and mitochondrial dysfunction contribute to the loss of dopaminergic neurons in PD. Sulfuretin is a potent antioxidant that is reported to be beneficial in the treatment of neurodegenerative diseases. In this study, we examined the protective effect of sulfuretin against 1-methyl-4-phenyl pyridinium (MPP^+^)-induced cell model of PD in SH-SY5Y cells and the underlying molecular mechanisms. Sulfuretin significantly decreased MPP^+^-induced apoptotic cell death, accompanied by a reduction in caspase 3 activity and polyADP-ribose polymerase (PARP) cleavage. Furthermore, it attenuated MPP^+^-induced production of intracellular reactive oxygen species (ROS) and disruption of mitochondrial membrane potential (MMP). Consistently, sulfuretin decreased p53 expression and the Bax/Bcl-2 ratio. Moreover, sulfuretin significantly increased the phosphorylation of Akt, GSK3β, and ERK. Pharmacological inhibitors of PI3K/Akt and ERK abolished the cytoprotective effects of sulfuretin against MPP^+^. An inhibitor of GSK3β mimicked sulfuretin-induced protection against MPP^+^. Taken together, these results suggest that sulfuretin significantly attenuates MPP^+^-induced neurotoxicity through Akt/GSK3β and ERK signaling pathways in SH-SY5Y cells. Our findings suggest that sulfuretin might be one of the potential candidates for the treatment of PD.

## 1. Introduction

Parkinson’s disease (PD) is the second most common neurodegenerative disease, clinically characterized by bradykinesia, rigidity, tremors, and abnormal posture [1]. The pathological feature of PD is the progressive degeneration of dopaminergic neurons in the substantia nigra pars compacta, causing a decrease in dopamine levels. Although molecular mechanisms responsible for neuronal death are not fully understood, oxidative stress and mitochondrial dysfunction are reported to drive PD pathogenesis [2].

The well-known neurotoxin, 1-methyl-4-phenyl pyridinium (MPP^+^), is an active metabolite of 1-methyl-4-phenyl-1,2,3,6-tetrahydropyridine (MPTP). It is highly toxic to dopaminergic neurons and is used to establish various in vitro and in vivo experimental models of PD [3,4]. MPP^+^ enters nigrostriatal neurons via dopamine transporters [5], and is transported into the mitochondria by the membrane potential [6]. MPP^+^-mediated neurotoxicity is triggered by oxidative stress [7] and apoptosis [8]. Associated signaling events include the inactivation of pro-survival phosphoinositide-3-kinase (PI3K/Akt) cascade [9,10] and the dysregulation of mitogen-activated protein kinases (MAPKs) [11]. PI3K/Akt signaling pathway is essential for protecting neuronal cells from oxidative stress [12]. Extracellular-signal-regulated kinase (ERK) plays a key role in cell proliferation, differentiation, survival, and apoptosis [13]. p53 also contributes to MPTP-induced neuronal apoptosis [14,15,16]. Since human neuroblastoma SH-SY5Y cells resemble dopaminergic neurons, they are extensively used to study PD-related neurotoxicity and mechanisms of neuroprotection [8].

*Rhus verniciflua* Stokes is used as a traditional medicine in East Asian countries, including Korea, China, and Japan, for the treatment of gastritis, stomach cancer, and atherosclerosis [17]. Sulfuretin is an antioxidant flavonoid, majorly isolated from the stem bark of the heartwood of *R. verniciflua* [18]. Sulfuretin exerts several pharmacological effects, including anticancer [19], anti-platelet [20], anti-inflammatory [21], antidiabetic [22], anti-mutagenic [23], anti-rheumatoid arthritis [24], and neuroprotective effects [25,26]. It was recently reported that sulfuretin also protected against 6-hydroxydopamine (6-OHDA)-induced neurotoxicity in an in vitro model of PD [25]. However, its protective effects against MPP^+^-induced oxidative stress and the subsequent apoptosis in SH-SY5Y cells has not been studied. In this study, we investigated the protective effects of sulfuretin against MPP^+^-induced cytotoxicity in SH-SY5Y cells, and identified the possible molecular mechanisms underlying these effects.

## 2. Results

### 2.1. Sulfuretin Protects SH-SY5Y Cells from MPP^+^-Induced Cytotoxicity

**Figure 1:**
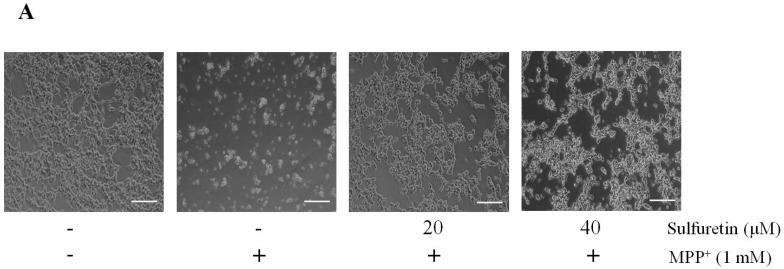

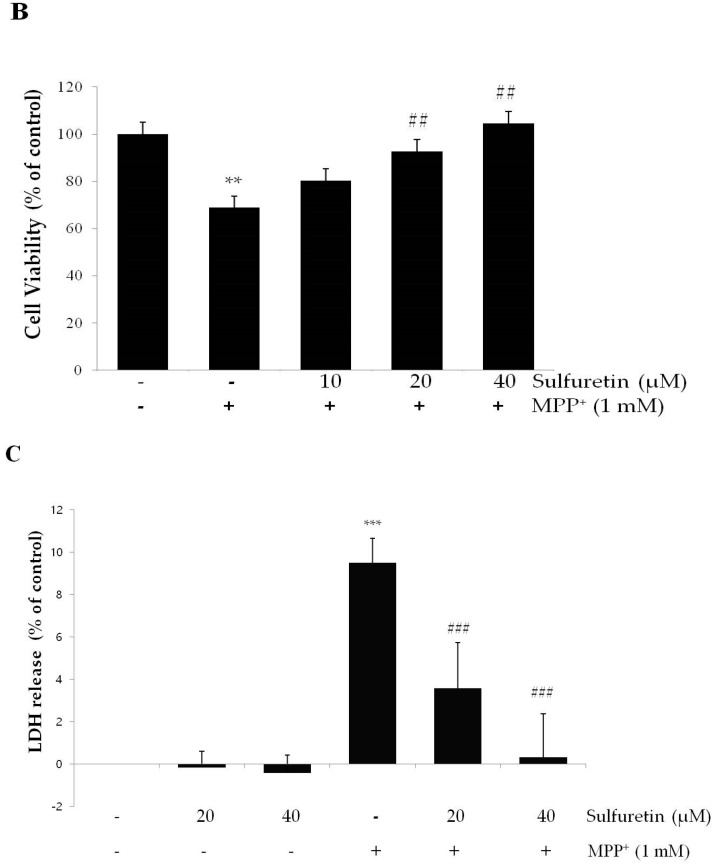
Sulfuretin protects SH-SY5Y cells against MPP^+^-induced cytotoxicity. Cells were pretreated with different doses of sulfuretin (10–40 μM) for 2 h and then exposed to MPP^+^ (1 mM) for 2 h. (**A**) After treatment, morphological changes were observed under a light microscope. Scale bar = 50 μm. Representative images are shown (*n* = 3). (**B**) Cell viability was measured using MTT assay. (**C**) Cytotoxicity was determined by measuring LDH release into the medium. Values are calculated using the equation as shown in Materials and Methods and presented relative to control as mean percentage change ± standard deviation (S.D.) (*n* = 5). Differences are statistically significant at ** *p* ˂ 0.01 and *** *p* ˂ 0.001 vs. the control group and ## *p* < 0.01 and ### *p* ˂ 0.001 vs. the MPP^+^ group.

Initially, we determined the effect of sulfuretin against MPP^+^-induced toxicity on the viability of SH-SY5Y cells. The cells were pretreated with sulfuretin (10–40 µM) for 2 h, followed by incubation with MPP^+^ (1 mM) for 24 h. We observed morphological changes that were associated with cell death, such as cell shrinkage and rounding up of cell bodies, in the MPP^+^-treated cells (Figure 1A). However, sulfuretin pretreatment markedly attenuated the morphological damage caused by MPP^+^. We also observed a significantly reduced cell viability in SH-SY5Y cells exposed to MPP^+^ (1 mM) compared to that in control cells (Figure 1B) (** *p <* 0.01). However, pretreatment with sulfuretin (20 or 40 µM) significantly increased cell viability in a dose-dependent manner. The treatment with 40 µM sulfuretin almost completely recovered the MPP^+^-induced loss in cell viability. Based on this result, sulfuretin at doses of 20 and 40 µM were evaluated further. Results of the lactate dehydrogenase (LDH) release assay were similar to those of the MTT assay; sulfuretin effectively inhibited LDH release into the culture medium, indicating reduced cytotoxicity (Figure 1C). 

### 2.2. Sulfuretin Suppresses MPP^+^-Induced Apoptosis, Accompanied by the Reduction of Caspase 3 Activity and PARP Proteolysis

**Figure 2:**
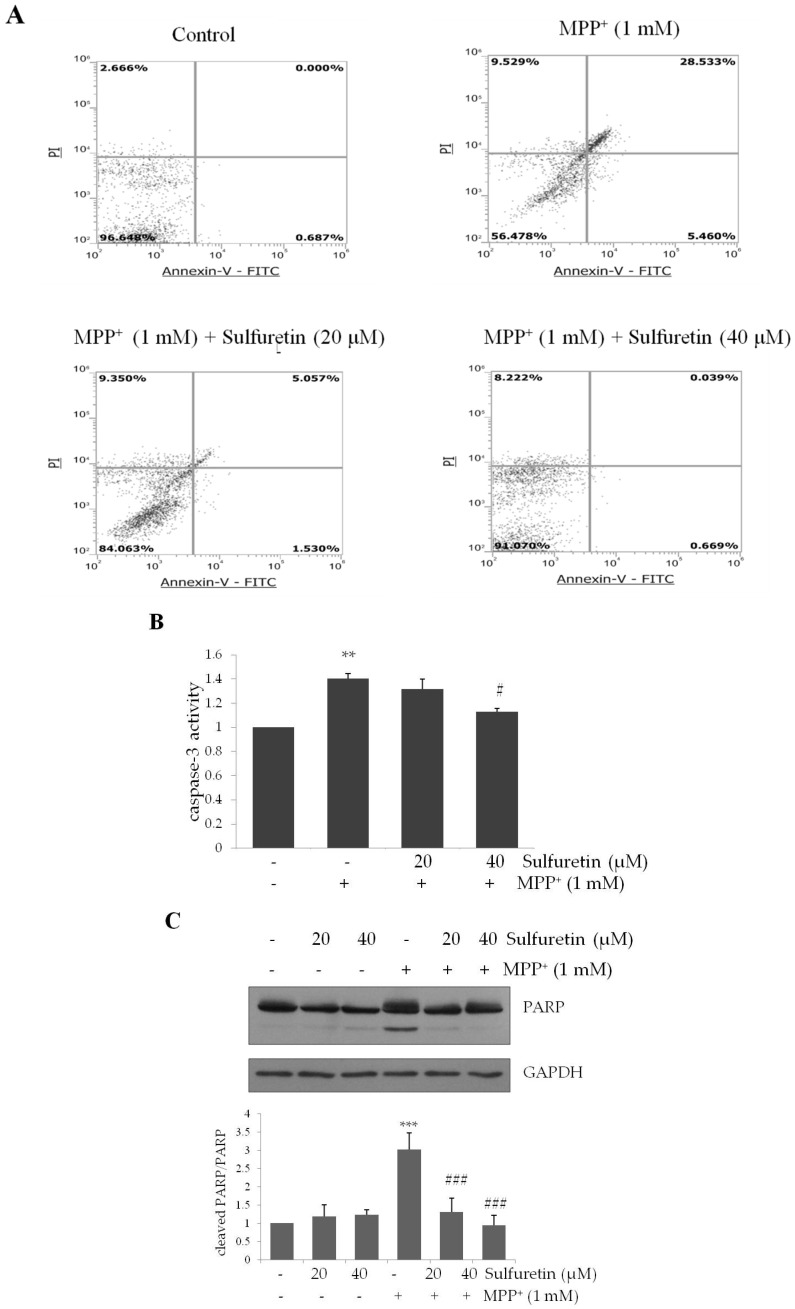
Sulfuretin suppresses MPP^+^-induced apoptosis, caspase-3 activity, and PARP proteolysis. SH-SY5Y cells were pretreated with sulfuretin for 2 h and then treated with MPP^+^ (1 mM) for 24 h. (**A**) Apoptosis was evaluated by annexin V and PI staining. Flow cytometric profile represents annexin V-FITC on the *x*-axis and PI on the *y*-axis. (**B**) Caspase 3 activity was measured by using colorimetric caspase-3 assay kit. (**C**) Protein levels of PARP were measured by western blot analysis. Representative blots and their densitometric quantification are shown. Values are presented relative to control as mean fold change ± S.D. (*n* = 3). Differences are statistically significant at ** *p* < 0.01 and *** *p* < 0.001 vs. the control group and # *p* ˂ 0.05 and ### *p* ˂ 0.001 vs. the MPP^+^ group.

We further confirmed the effect of sulfuretin on MPP^+^-induced apoptosis in SH-SY5Y cells using flow cytometry analysis with annexin V and PI double-staining. The annexin V(−)/PI(−), annexin V(+)/PI(−), and annexin V(+)/PI(+) populations indicate healthy, early apoptotic, and late apoptotic cells, respectively. As illustrated in Figure 2A, MPP^+^ increased the rate of apoptosis in SH-SY5Y cells, which was reversed by pretreatment with sulfuretin (40 µM). In MPP^+^-treated cells, the percentage of apoptosis (34%) was significantly higher than that in control cells. In contrast, pretreatment with sulfuretin at 20 and 40 µM markedly reduced the rate of apoptosis to 6.587% and 0.708%, respectively, compared to that in MPP^+^-treated cells (** *p* < 0.01). These results suggest that sulfuretin protects against MPP^+^-induced apoptosis in SH-SY5Y cells.

Caspase 3 activation and PARP cleavage are important biomarkers of apoptosis. While MPP^+^ treatment increased caspase 3 activity, pretreatment with sulfuretin significantly attenuated MPP^+^-induced caspase 3 activation (Figure 2B). Activated caspase 3 cleaves full-length PARP (116 kDa) nuclear protein to a PARP fragment (85 kDa). PARP proteolysis was significantly enhanced following treatment with MPP^+^ (1 mM); however, sulfuretin pretreatment completely attenuated MPP^+^-induced PARP proteolysis (Figure 2C). This suggests that sulfuretin provides neuroprotection by inhibiting apoptosis.

### 2.3. Sulfuretin Reverses MPP^+^-Induced Intracellular Accumulation of ROS and Reduction of Mitochondrial Membrane Potential (MMP)

**Figure 3:**
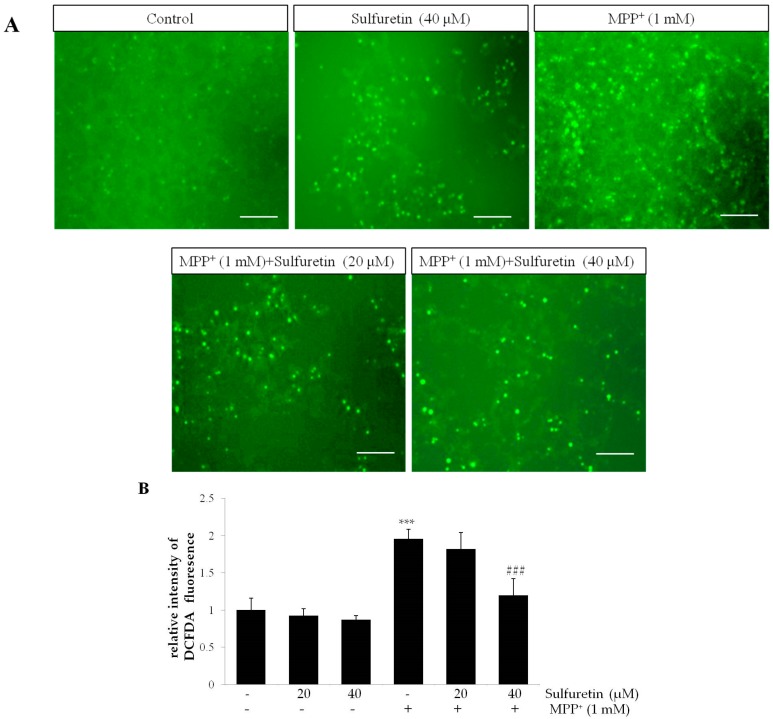

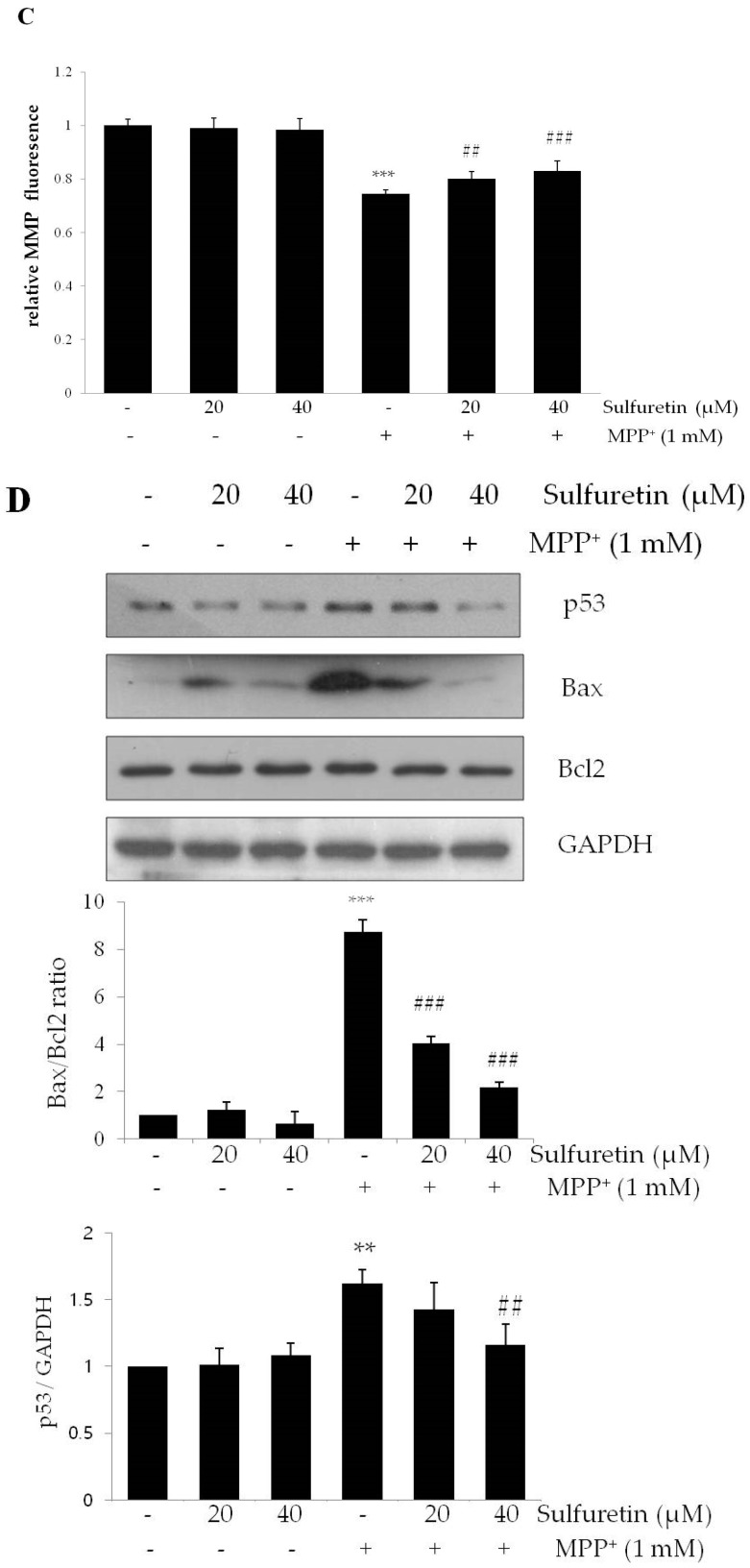
Sulfuretin reverses MPP^+^-induced intracellular accumulation of ROS and reduction in MMP. SH-SY5Y cells were pretreated with sulfuretin (20 or 40 μM) for 2 h and then exposed to MPP^+^ (1 mM) for 24 h. Further, the cells were incubated for 30 min at 37 °C with 2,7-dichlorofluorescein diacetate (DCFH-DA) (10 μM). (**A**) Representative images of the cells under a fluorescence microscope are shown. DCFH-DA oxidation by ROS is indicated in green colour. Scale bar = 50 μM. (**B**) DCFH-DA fluorescence intensities were measured by fluorimetry with a plate reader at ex/em: 485/535 nm. (**C**) MMP was measured by fluorimetry with a plate reader at ex/em: 549/575 nm. (**D**) Protein levels of p53, Bax, and Bcl-2 were measured by Western blot analysis. Representative blots and their densitometric quantification are shown. Values are presented relative to control as mean fold change ± S.D. (*n* = 3). Differences are statistically significant at ** *p* < 0.01, *** *p* < 0.001 vs. the control group and ## *p* < 0.01, ### *p* < 0.001 vs. the MPP^+^ group.

ROS and mitochondrial dysfunction drive MPP^+^-induced neuronal apoptosis. We examined whether the protective effect of sulfuretin against MPP^+^-induced cytotoxicity is mediated by an antioxidant mechanism. Intracellular ROS levels were measured by 2′,7′-dichlorodihydrofluorescein diacetate (DCFH-DA) fluorescence assay. Exposure to MPP^+^ (1 mM) for 24 h significantly increased the intracellular ROS levels in SH-SY5Y cells. In contrast, sulfuretin pretreatment significantly suppressed the intracellular ROS accumulation induced by MPP^+^ (Figure 3A,B).

MMP is a marker of mitochondrial function, which is sensitive to oxidative stress and apoptosis [27]. We evaluated the effect of sulfuretin against MPP^+^-induced reduction in MMP. MPP^+^ significantly reduced MMP, whereas pretreatment with sulfuretin significantly attenuated this reduction in SH-SY5Y cells (Figure 3C). These results indicate that sulfuretin efficiently suppresses MPP^+^-induced oxidative stress and recovers the MMP reduced by MPP^+^ in SH-SY5Y cells.

Because an increase in p53 expression and Bax/Bcl-2 ratio is associated with MMP disruption and mitochondrial dysfunction, we measured the expression of p53, Bax, and Bcl-2 proteins. MPP^+^ treatment significantly increased the expression of p53 and its downstream target Bax, which was prevented by sulfuretin treatment. However, neither MPP^+^ nor sulfuretin significantly altered the Bcl-2 protein level (Figure 3D). MPP^+^ increased the Bax/Bcl-2 ratio, which was prevented by sulfuretin pretreatment. These results indicate that sulfuretin restores the balance between anti-apoptotic and pro-apoptotic proteins, preserves mitochondrial function, and promotes cell survival.

### 2.4. Sulfuretin Regulates Akt/GSK3β and ERK Signaling Pathways

**Figure 4:**
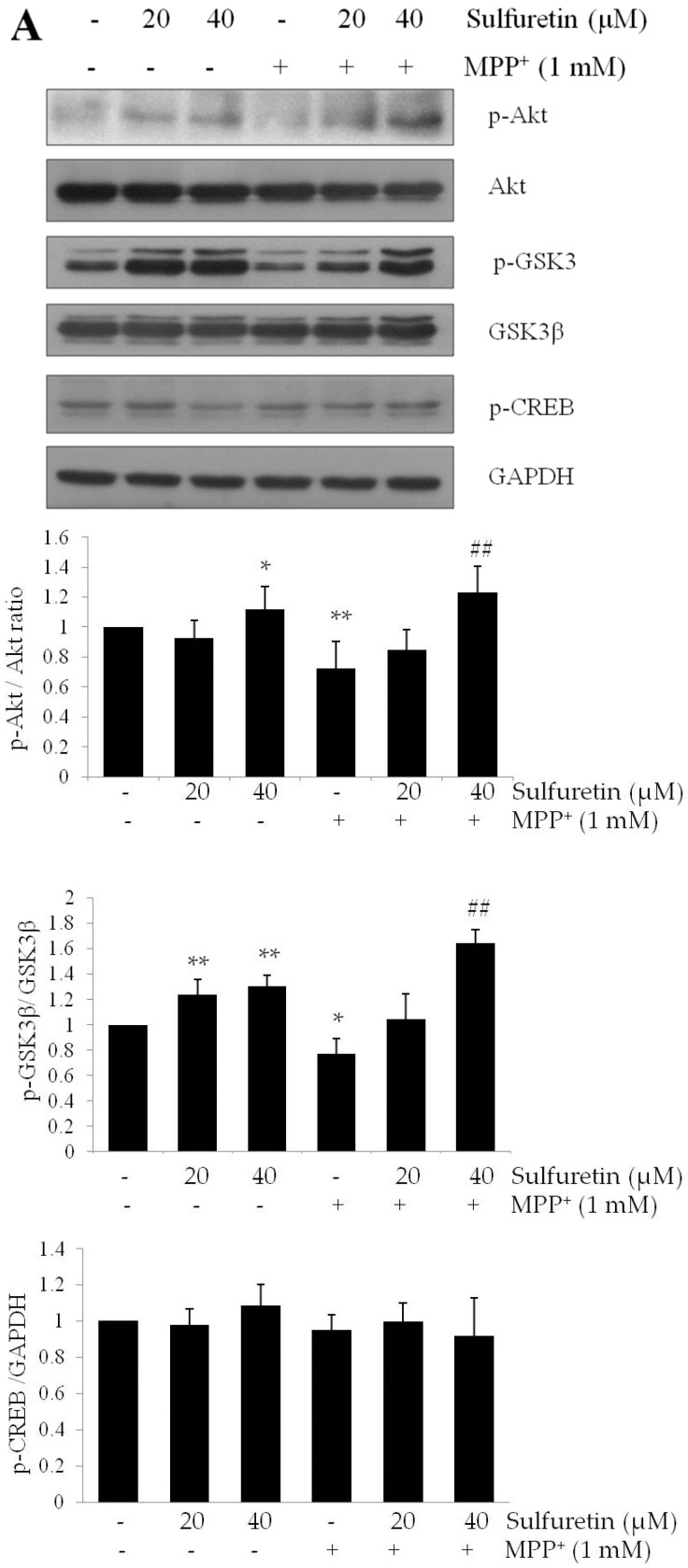

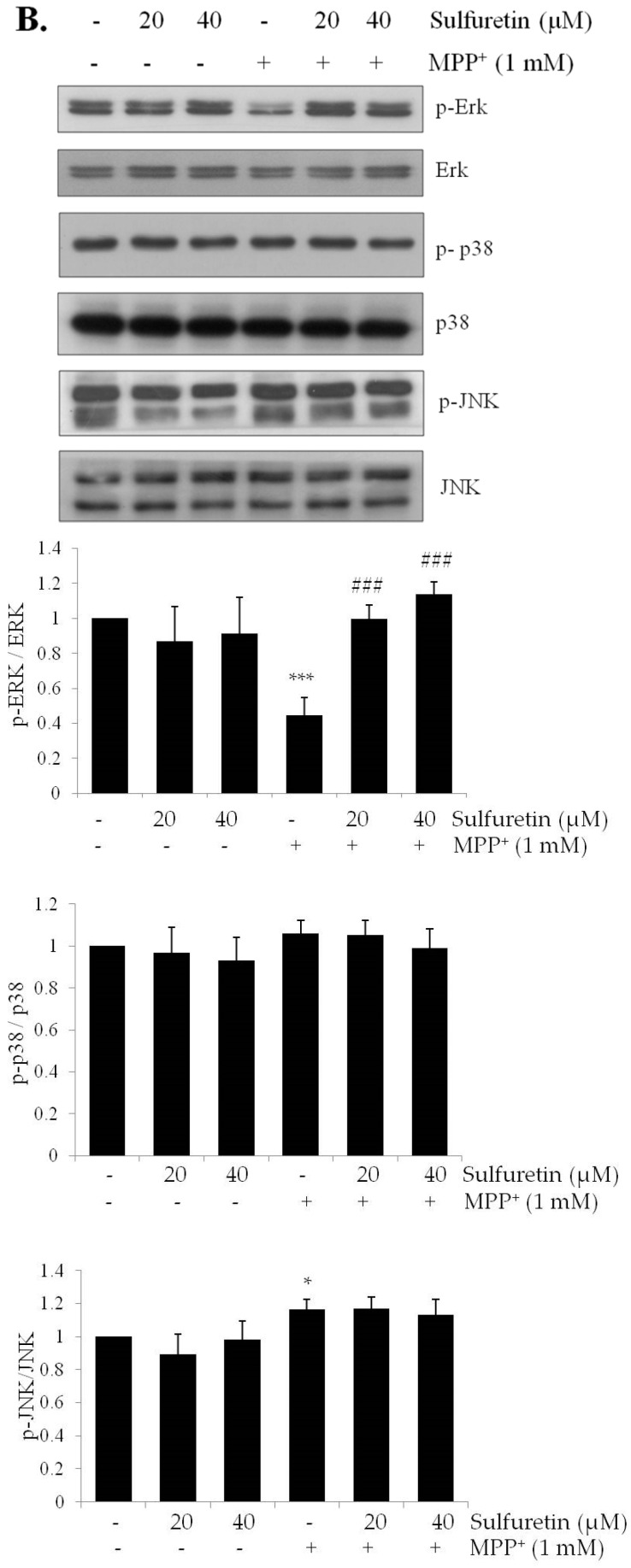
MPP^+^ decreases Akt/GSK3β and ERK phosphorylation and increases p53 expression, whereas sulfuretin reverses its effect. SH-SY5Y cells were pretreated with sulfuretin for 2 h and then treated with MPP^+^ for 24 h. After cell lysis, the extracted proteins were subjected to Western blot analysis using specific antibodies. Protein levels of (**A**) p-Akt, Akt, p-GSK3β, GSK3β, p-CREB, and GAPDH; (**B**) p-ERK, ERK, p-p38, p38, p-JNK, and JNK were determined. Representative blots and their densitometric quantification are shown. Values are presented relative to control as mean fold change ± S.D. (*n* = 3). Differences are statistically significant at * *p <* 0.05, ** *p <* 0.01, and *** *p <* 0.001 vs. the control group and ## *p <* 0.01, and ### *p <* 0.001 vs. the MPP^+^ group.

Defective PI3K/Akt pathway signaling leads to PD-like neurodegeneration, and the activation of Akt may provide protection against neurodegenerative diseases [28]. MPP^+^ is reported to inhibit the PI3K/MAPK pathway [29]. To elucidate the molecular mechanisms underlying the protective effect of sulfuretin against MPP^+^, we evaluated the PI3K/MAPK signaling pathway by Western blot analysis. As shown in Figure 4A,B, we found that MPP^+^ markedly decreased Akt, GSK3β, and ERK phosphorylation, whereas pretreatment with sulfuretin reversed this decrease in phosphorylation by MPP^+^. Treatment with sulfuretin alone also increased the phosphorylation of Akt, GSK3β, and ERK. However, sulfuretin did not affect the phosphorylation of JNK and p38 (Figure 4B). These findings suggest that sulfuretin might prevent MPP^+^-induced cytotoxicity through the activation of Akt/GSK3β and ERK.

### 2.5. PI3K/Akt and MAPK Inhibitors Suppress the Neuroprotective Effects of Sulfuretin against MPP^+^

**Figure 5:**
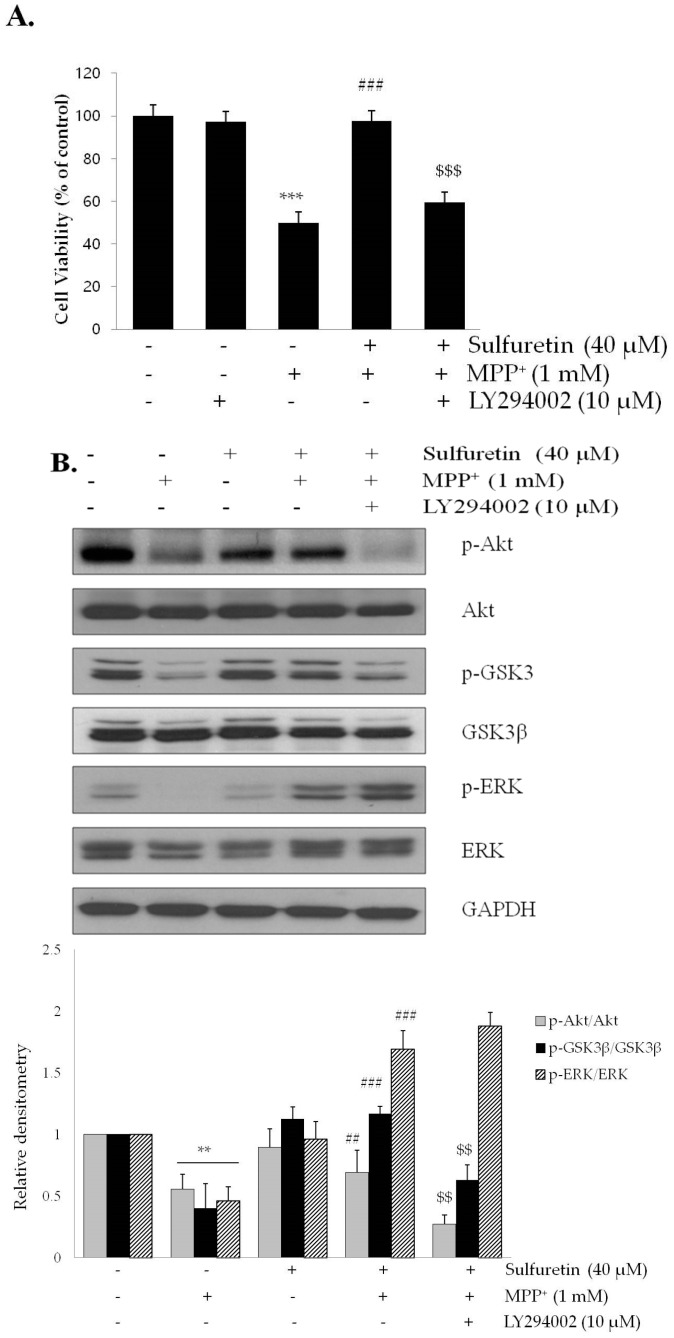

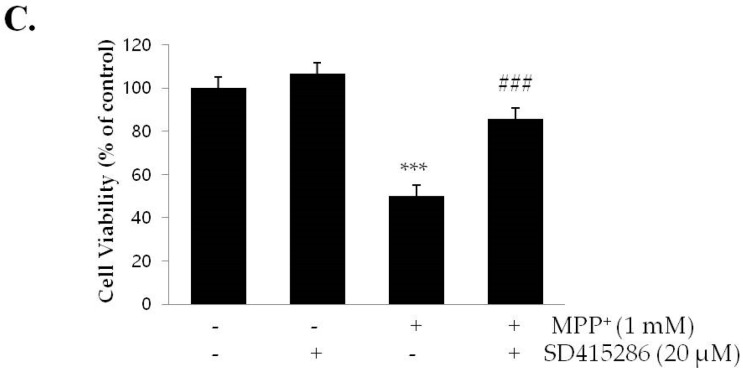
LY294002 suppresses sulfuretin-induced protection against MPP^+^, whereas SB415286 reverses MPP^+^-induced cytotoxicity. SH-SY5Y cells were pretreated with or without LY294002 (10 μM) for 2 h, followed by treatment with or without sulfuretin (40 μM) for 2 h and exposed to MPP^+^ (1 mM) for 24 h. (**A**) Cell viability was measured by MTT assay. Values are presented relative to control as mean percentage change ± S.D. (*n* = 3). (**B**) Protein levels of p-Akt, Akt, p-GSK3β, GSK3β, p-ERK, ERK, and GAPDH were determined by Western blot analysis. Representative blots and their densitometric quantification are shown. Values are presented relative to control as mean fold change ± S.D. (*n* = 3). (**C**) SH-SY5Y cells were pretreated with or without SB415286 (20 μM) for 2 h, and then exposed to MPP^+^ (1 mM) for 24 h. Cell viability was measured by MTT assay. Values are presented relative to control as mean percentage change ± S.D. (*n* = 3). Differences are statistically significant at ** *p <* 0.01, *** *p <* 0.001 vs. the control group, ## *p <* 0.01, ### *p <* 0.001 vs. the MPP^+^ group, and $$ *p <* 0.01, $$$ *p <* 0.001 vs. the MPP^+^ and sulfuretin co-treated group.

We further examined the involvement of PI3K/Akt/GSK3β and ERK signaling pathway in sulfuretin-induced protection against MPP^+^. The cells were pretreated with a PI3K/Akt inhibitor, LY294002, and sulfuretin, followed by MPP^+^ treatment for 24 h. Results of the MTT assay showed that LY294002 abolished sulfuretin-induced protection against MPP^+^ cytotoxicity (Figure 5A). Moreover, western blot analysis showed that LY294002 decreased the phosphorylation of GSK3β as well as of Akt, suggesting that GSK3β is a downstream signaling molecule of Akt (Figure 5B). Conversely, ERK phosphorylation was not affected by LY294002, indicating an independent signaling pathway. Increased phosphorylation of GSK3β at Ser9 induced by Akt leads to GSK3β inactivation [30,31]. The GSK3β inhibitor, SB415286, markedly prevented neuronal death induced by MPP^+^, which mimicked the protective effects of sulfuretin (Figure 5C). These data suggest that sulfuretin-induced protection against MPP^+^ cytotoxicity might be mediated by Akt activation and subsequent GSK3β inactivation.

**Figure 6:**
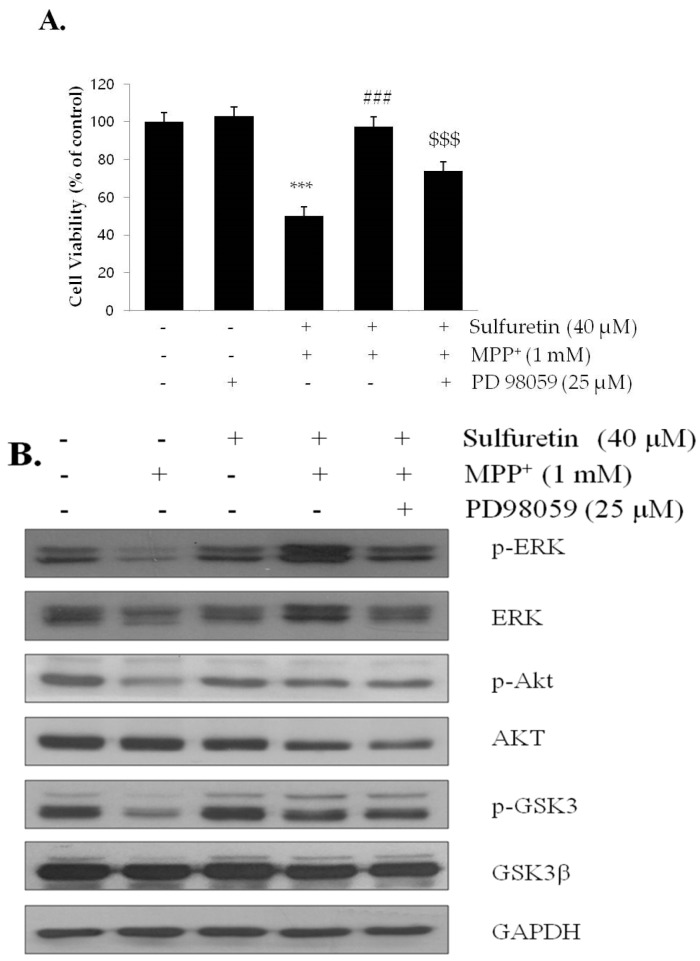

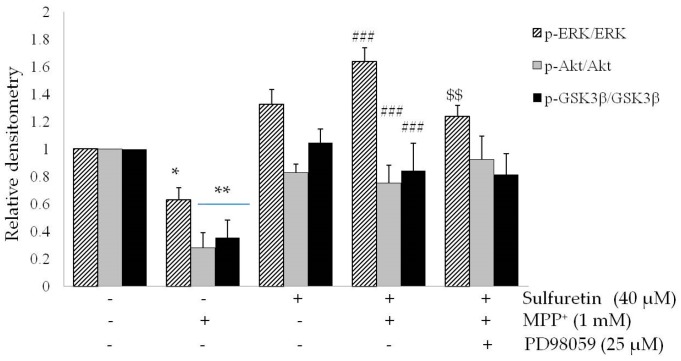
PD98059 suppresses sulfuretin-induced protection against MPP^+^. SH-SY5Y cells were pretreated with or without PD98059 (10 μM) for 2 h, followed by treatment with or without sulfuretin (40 μM) for 2 h, and exposed to MPP^+^ (1 mM) for 24 h. (**A**) Cell viability was measured by MTT assay. Values are presented relative to control as mean percentage change ± S.D. (*n* = 3). (**B**) Protein levels of p-ERK, ERK, p-Akt, Akt, p-GSK3β, GSK3β, and GAPDH were determined by Western blot analysis. Representative blots and their densitometric quantification are shown. Values are presented relative to control as mean fold change ± S.D. (*n* = 3). Differences are statistically significant at * *p <* 0.05, ** *p <* 0.01, *** *p <* 0.001 vs. the control group, ### *p <* 0.001 vs. the MPP^+^ group, and $$ *p <* 0.01, $$$ *p <* 0.001 vs. the MPP^+^ and sulfuretin pretreated group.

In addition, the protective effect of sulfuretin was abolished in the presence of the MAPK inhibitor, PD98059 (Figure 6A), suggesting a critical role of ERK in sulfuretin-induced cytoprotection. PD98059 significantly abrogated ERK phosphorylation; however, it did not affect the phosphorylation of Akt and GSK3β (Figure 6B). Taken together, these data suggest that sulfuretin-induced protective effects by Akt/GSK3β and ERK pathways are independent of each other.

## 3. Discussion

Naturally occurring flavonoids are polyphenols, found ubiquitously in plants [32]. Interestingly, many flavonoid compounds exhibit neuroprotective effects in humans [33]. In vitro experiments have reported that sulfuretin, as a flavonoid, provides neuroprotection against amyloid β and 6-OHDA [25,26]. In addition, sulfuretin attenuates neuroinflammation in microglial cells [34]. Like 6-OHDA, MPP^+^ is used as a neurotoxin to induce dopaminergic neuronal cell death associated with PD. However, the effects of sulfuretin against MPP^+^-induced neurotoxicity are still unknown. In this study, we evaluated the anti-Parkinson’s effects of sulfuretin using MPP^+^-treated SH-SY5Y cells and elucidated its molecular mechanisms. We showed that sulfuretin exerted a protective effect against MPP^+^-induced cytotoxicity in SH-SY5Y cells. Furthermore, sulfuretin suppressed intracellular ROS accumulation and prevented mitochondrial dysfunction. Moreover, the sulfuretin-induced neuroprotective effects were mediated by PI3K/Akt/GSK3β and ERK pathways.

MPP^+^ is a neurotoxin that selectively damages catecholaminergic neurons, including dopaminergic neurons, and is widely accepted as an experimental model of PD in vitro [35,36,37]. MPP^+^ increases the mitochondrial outer-membrane permeability, leading to increased cytosolic cytochrome C and apoptotic proteins [38]. Cytosolic cytochrome C forms an apoptosome with the apoptosis-activating factor, which leads to the activation of caspases 9 and 3 [39]. PARP is an enzyme involved in apoptosis as a downstream target of caspase 3. It is abundantly present in the nucleus and normally functions as a DNA repair enzyme [40,41]. Proteolytic cleavage of PARP occurs during catechol-thioether-induced apoptosis in human SH-SY5Y neuroblastoma cells [42] and MPP^+^-induced apoptosis in cerebellar granule neurons [43]. Consistently, we observed that MPP^+^ treatment increases caspase 3 activity and PARP cleavage (Figure 2), along with significant apoptotic cell death in SH-SY5Y cells (Figure 1). 

However, sulfuretin ameliorated MPP^+^-induced apoptosis, accompanied by the reduction of caspase 3 activity and PARP cleavage in SH-SY5Y cells (Figure 2). Sulfuretin is reported to have a protective effect against 6-OHDA in SH-SY5Y cells [25]. Considering that both 6-OHDA and MPP^+^ are widely used to induce PD-like neurodegeneration, it is likely that sulfuretin has a therapeutic potential in PD induced by various neurotoxins. Both 6-OHDA and MPP^+^ are selectively toxic to dopaminergic neurons. They act as high-affinity substrates for the dopamine reuptake system and express their cytotoxicity through ROS production [44]. Our study also showed that MPP^+^ significantly increases ROS production and sulfuretin treatment effectively attenuates this effect of MPP^+^ (Figure 3A,B). Consistently, a previous study demonstrated that sulfuretin decreases 6-OHDA-induced ROS production and increases the activities of antioxidant enzymes, such as superoxide dismutase, catalase, and glutathione in SH-SY5Y cells [25]. These data clearly suggest that sulfuretin has a potent antioxidant effect, which might be a common molecular mechanism underlying the protective effects of sulfuretin against PD-associated insults. Oxidative damage occurs in the PD brain [45], and overproduction of ROS can impair cellular functions to trigger apoptotic mechanisms in PD [46]. MPP^+^-induced oxidative stress opens the mitochondrial permeability transition pore that decreases the MMP [36,47]. In addition, MPP^+^ increases the Bax/Bcl-2 ratio, and Bax translocation to the mitochondrial membrane further decreases the MMP; Bcl-2 inhibits Bax translocation [8,48,49]. Consistently, our data showed that treatment with MPP^+^ significantly increased the Bax/Bcl-2 ratio and decreased the MMP and sulfuretin co-treatment effectively prevented MPP^+^-induced changes in Bax/Bcl-2 ratio and MMP in SH-SY5Y cells (Figure 3C,D).

The transcription factor, p53, modulates a wide range of cellular process, including cell cycle progression, DNA repair, apoptosis, and cellular stress response [50,51]. Activated p53 is responsible for dopaminergic neuronal death, as shown in models of MPTP-induced PD [15,52]. p53 inhibition is reported to be highly effective in reducing dopaminergic neuronal death and in preventing motor dysfunction in a mouse model of PD [53]. In addition, overproduction of ROS activates p53, leading to further DNA damage [54]. In particular, p53 directly induces the expression of the pro-apoptotic protein, Bax, and directly inhibits the anti-apoptotic protein, Bcl-2 [55]. Consistent with previous reports [56,57], we observed that MPP^+^ treatment increases the protein expression of p53 and its downstream target, Bax (Figure 3D). Furthermore, sulfuretin attenuated the expression of p53 and Bax in a dose-dependent manner. These results suggest that sulfuretin might reduce Bax expression through p53 regulation, thereby exhibiting an anti-apoptotic effect.

Phosphorylation of PI3K/Akt and GSK3β is a key step in various cellular processes, such as proliferation, growth, survival, and apoptosis [58,59]. Previous studies have demonstrated that MPP^+^ rapidly and reversibly decreases Akt and GSK3β phosphorylation [31,60], which correlates with increased neuronal death [61,62]. Therefore, we evaluated whether PI3K/Akt and GSK3β signaling pathways are involved in the anti-apoptotic effects of sulfuretin. Consistent with previous reports, MPP^+^ decreased the phosphorylation of Akt at Ser473 and GSK3β at Ser9; however, sulfuretin reversed the dephosphorylation of Akt and GSK3β in MPP^+^-treated SH-SY5Y cells (Figure 4A). GSK3β is a downstream target of Akt [63] and an important mediator of MPP^+^-induced cell injury [64]. GSK3β activation facilitates mitochondrial dysfunction, whereas its inhibition prevents neuronal loss by suppressing pro-apoptotic proteins [65]. Phosphorylation of GSK3β at Ser9 is mainly controlled by Akt and this phosphorylation significantly inhibits the activity of GSK3β [66]. LY294002 abolished the anti-apoptotic effect of sulfuretin by preventing the phosphorylation of Akt and GSK3β (Figure 5A,B). Furthermore, SB415286 attenuated MPP^+^-induced apoptosis, mimicking the protective effects of sulfuretin in SH-SY5Y cells (Figure 5C). These results demonstrated that PI3K/Akt and GSK3β mediates the protective effects of sulfuretin against MPP^+^ in SH-SY5Y cells. Consistent with our results, it was reported that PI3K/Akt is activated by sulfuretin and responsible for th sulfuretin-induced protective effect against amyloid β [26].

MAPK signaling pathways are involved in many cellular events, including differentiation, proliferation, and apoptosis, and at least three major MAPK subfamilies (ERK, JNK, and p38) have been characterized [67]. Among them, ERK increases the survival of dopaminergic neurons [35,68]. The phosphorylation of ERK is reported to be suppressed after 4 h of exposure to MPP^+^ in SH-SY5Y cells [35]. Our study confirmed that MPP^+^ reduced the phosphorylation of ERK, whereas sulfuretin reversed the MPP^+^-mediated ERK dephosphorylation (Figure 4B). It has previously been demonstrated that phosphorylated ERK migrates to the nucleus and regulates various transcription factors, leading to changes in gene expression and cell proliferation [69]. In particular, PD98059 abolished the protective effects of sulfuretin on cell viability, suggesting that ERK is critical for sulfuretin-induced protection against MPP^+^ cytotoxicity (Figure 6A). Interestingly, LY294002 decreased the phosphorylation of Akt and GSK3β without altering ERK phosphorylation. Consistently, PD98059 decreased ERK phosphorylation without changing phosphorylation of Akt or GSK3β (Figure 6B). These results indicate that both Akt/GSK3β and ERK contribute to the protective effects of sulfuretin in a mutually independent manner. Similarly, Zhang et al. showed that in primary dopaminergic neurons, valproic acid has protective effects against MPP^+^-induced neurotoxicity such as apoptosis, dopamine uptake reduction and tyrosine hydroxylase inactivation [29]. LY294002 and PD98059 reversed valproic acid-induced neuroprotective effects. Interestingly, pretreatment with both LY294002 and PD98059 showed further reverse effects compared to LY294002 or PD98059 alone, suggesting additive effect of PI3K/Akt and ERK signaling pathways.

Although we did not investigate how sulfuretin affects these signaling pathways, ROS might have a potential as an upstream molecules regulating both signaling pathways. Previous paper demonstrated that MPTP-induced oxidative stress oxidizes the critical cysteines in Akt and this modified Akt is associated with PP2A which leads to dephosphorylation of Akt [70]. In the paper, thiol antioxidants attenuated MPTP-induced loss of Akt phosphorylation in mouse midbrain, indicating the critical role of ROS in MPP^+^-induced Akt dephosphorylation. In case of ERK, it was reported that MPP^+^ increased α-synuclein expression through ROS production and the increased α-synuclein expression inactivated ERK, thereby increasing caspase 3 activation [71]. We previously reported that glucose deficiency increased ROS production and decreased ERK phosphorylation in SH-SY5Y cells [72]. Interestingly, ROS inhibitor clearly recovered ERK phosphorylation decreased by glucose deficiency, suggesting ROS-induced downregulation in ERK phosphorylation. The present study also showed that sulfuretin reduced MPP^+^-induced ROS production (Figure 3). Based on these studies, ROS might be an upstream molecular mechanism regulating both PI3K/Akt and ERK signaling pathways. However, further studies will be needed to clarify this issues.

Unexpectedly, sulfuretin regulated some signaling molecules in a way that is different from a previous report [25]. Sulfuretin attenuates ROS production induced by both MPP^+^ and 6-OHDA. However, it was reported to decrease 6-OHDA-induced phosphorylation of Akt and GSK3β and not to alter ERK phosphorylation in SH-SY5Y cells, which is in contrast to our data. Although sulfuretin was reported to decrease the phosphorylation of p38 and JNK induced by 6-OHDA, we did not observe a change in the phosphorylation of p38 and JNK in our study. It is likely that these discrepancies are attributable to the different neurotoxins, MPP^+^ and 6-OHDA. Although both neurotoxins increase ROS, 6-OHDA produces ROS through redox cycling, which involves one-electron reduction of oxygen, resulting in superoxide and semiquinone radical intermediates [73]. MPP^+^ is generated from MPTP through the action of glial monoamine oxidase B [74]. It accumulates within the mitochondria of dopaminergic neurons and irreversibly inhibits complex I of the mitochondrial respiratory chain, thereby increasing ROS production and causing an acute ATP deficiency [75,76]. These differences between MPP^+^ and 6-OHDA in ROS production might account for the differences in the regulation of their signaling pathways by sulfuretin. However, further studies are needed.

## 4. Materials and Methods

### 4.1. Materials

Dulbecco’s modified Eagle medium (DMEM) and fetal bovine serum (FBS) were purchased from Gibco (Carlsbad, CA, USA). LY294002, SB415286, DCFH-DA and MPP^+^ were purchased from Sigma Chemicals (St. Louis, MO, USA). PD98059, Anti-phospho-p44/42 MAPK (ERK1/2, Thr202/Tyr204), anti-ERK, anti-phospho-GSK3 β (Ser9), anti-GSK3β, anti-phospho-Akt (Ser473), anti-Akt, anti-phospho-JNK, anti-JNK, anti-phospho-p38, anti-p38 and anti-phospho-CREB (Ser133) were obtained from Cell Signaling Technology (Boston, MA, USA). The antibody against GAPDH and secondary antibodies were purchased from Santa Cruz Biotechnology (Santa Cruz, CA, USA). 

### 4.2. Identification and Isolation of Sulfuretin

Sulfuretin was isolated from *R. verniciflua* Stokes, as described in detail in our previous report [77]. In brief, the leaves were dried and extracted with methanol by heating at 40 °C. After solvent evaporation in a rotary evaporator and the resulting powder was suspended in water and then fractionated with butanol. The fractioned sample was run on a silica gel and eluted with CHCl3–MeOH (100%→50%) to obtain fractions F1–F10. F6 was further subjected to reversed-phase silica gel column chromatography using water–MeOH (50%→70%) as eluent. Pure sulfuretin (orange amorphous powder) was isolated and characterized using spectroscopic methods (UV absorption, ^1^H- and ^13^C-NMR).

### 4.3. Cell Culture

Human neuroblastoma SH-SY5Y cells were purchased from the Korean Cell Line Bank (Seoul, Korea). The cells were cultured in DMEM with 10% FBS and maintained in a humidified atmosphere of CO_2_ (5%) at 37 °C. They were seeded in 96-well plates and 6-well plates at densities of 1 × 10^5^ and 2 × 10^6^ cells, respectively. The cells, plated in 6-well plates, were pretreated with sulfuretin for 2 h and then exposed to MPP^+^. After 24 h, the cell morphology was observed using an optical microscope.

### 4.4. Cell Viability Assay

Cell viability was measured using the 3-(4,5-dimethylthiazol-2-yl)-2,5-diphenyl tetrazolium bromide (MTT) assay, as previously reported [78]. In brief, SH-SY5Y cells (1 × 10^5^ cells/well, passage numbers 9~11, 15~16) seeded in 96-well plates were pretreated with sulfuretin for 2 h and then treated with MPP^+^ for 24 h. Further, the MTT solution (1 mg/mL) was added into each well and the plates were incubated at room temperature for 2 h. Then, 100 μL of DMSO was added to each well to dissolve precipitate formazan crystals and the absorbance at 540 nm was measured with a microplate ELISA reader.

### 4.5. Lactate Dehydrogenase (LDH) Release

Cytotoxicity was quantitatively evaluated by examining the release of LDH in the medium. As high control, cells (passage numbers 13~14, 27~29) were pretreated for 1 h with 1% (*v*/*v*) Triton X-100 in the culture medium. All procedures were carried out as per the supplier’s instructions (LDH cytotoxicity Colorimetric Assay Kit, BioVision). In brief, after treatment, 100 µL of culture medium from each well was transferred to a new 96-well plate, 100 µL of reaction mixture was then added and incubated at room temperature for 30 min. The release of LDH was measured by noting the optical density (OD) at 490 nm using a microplate ELISA reader. LDH release into the medium was calculated using the following equation:

LDH release (%) = (OD value of treated well − OD value of non-treated control)/ (OD value of high control − OD value of non-treated control) × 100%

### 4.6. Flow Cytometric Analysis of Apoptosis

Flow cytometry was used to assess membrane and nuclear events during apoptosis, as previously described [79]. An apoptosis kit (Life Technologies, Grand Island, NY, USA), based on Alexa fluor^®^ 488-annexin V and propidium iodide (PI) for flow cytometry, was used to detect apoptotic and necrotic cells according to the manufacturer’s instructions. In brief, SH-SY5Y cells (passage numbers 18~21) were seeded in −6 well plates (2 × 10^6^ cells/well) and pretreated with sulfuretin for 2 h, followed by MPP^+^ (1 mM) for 24 h. As positive control, the cells were cultured with H_2_O_2_ (200 mM) for 30 min. After treatment, the cells were washed in cold phosphate buffered saline (PBS), centrifuged, and suspended in annexin-binding buffer. To each 100 µL of cell suspension, 5 µL of annexin V and 1 µL of PI (100 µg/mL) working solutions were added. SH-SY5Y cells were analyzed by flow cytometry with fluorescence detection (ex/em: 488/530 and 575 nm). The cells were considered positive on the basis of annexin V or PI fluorescence intensity. Positivity to annexin V occurs when release of phosphatidylserine, which indicates the early stage of apoptosis. Positivity to PI occurs when damage to the cell membrane, which indicates late stage of apoptosis, as well as necrosis. Apoptotic, non-viable, and viable cells were identified as annexin V(+)/PI(−), annexin V(+)/PI(+), and annexin V(−)/PI(−), respectively. Fluorescence intensities were analyzed using a flow cytometer (Cytomics™ FC 500, Beckman Coulter Inc., Brea, CA, USA). The experiment was repeated 3 times.

### 4.7. Intracellular ROS Measurement

Intracellular ROS production was evaluated using the DCFH-DA fluorescence probe. Cells (passage numbers 12~14, 27~28) were seeded in 96-well and 6-well plates, pretreated with sulfuretin for 2 h, and then treated with MPP^+^ (1 mM) for 24 h. Further, the cells were incubated with DCFH-DA (10 µM) for 30 min at 37 °C, and DCFH-DA fluorescence were visualized under a fluorescence microscope (Nikon-TS100-F, Tokyo, Japan) or scanned at Ex/Em: 485/535 nm with a plate reader (Wallac, PerkinElmer, Waltham, MA, USA).

### 4.8. Mitochondrial Membrane Potential (MMP) Measurement

Tetramethylrhodamine ethyl ester (TMRE) is a cell-permeant, positively-charged, red-orange dye, which accumulates within negatively-charged active mitochondria. Inactive mitochondria have a decreased membrane potential, within which TMRE does not accumulate. A TMRE-based MMP assay kit-F (Biovision, Milpitas, CA, USA) was used according to the manufacturer’s instructions. Briefly, 1 × 10^5^ cells (passage numbers 9~11) were seeded in 96-well plates and treated with sulfuretin in serum-free media, followed by treatment with MPP^+^ (1 mM) for 24 h. Further, the cells were incubated with MMP-sensitive fluorescent TMRE for 20 min at 37 °C and 5% CO_2_. As negative control, the cells were treated with FCCP (carbonyl cyanide-4-phenylhydrazone; 100 μM) for 15 min prior to incubation with TMRE. The fluorescence intensity was measured with a plate reader (Wallac, PerkinElmer, Waltham, MA, USA) at Ex/Em: 549/575 nm.

### 4.9. Colorimetric Assay of Caspase Activites

Caspase-3 activity in cultures were measured using the caspase 3 Colorimetric Assay Kit (K106-100, BioVision, CA, USA) according to the manufacturer’s protocol. In brief, the SH-SY5Y cells (passage numbers 12~13, 17~18) were seeded in 6 well plate and treated with sulfuretin for 2 h, followed by treatment with MPP^+^ (1 mM) for 24 h. Proteins were extracted with cells lysis buffer (provided with the kits), vertex and incubated on ice for 10 min. After centrifugation at 10,000× *g* for 1 min at 4 °C, the supernatants was collected. Equal amount of protein was exposed to a reaction mixture contained 50 µL of cell lysate and 5 µL of caspase-3 substrate (Ac-DEVD-pNA) in assay buffer. Reaction mixtures were incubated for 2 h at 37 °C and the absorption was measured at 405 nm.

### 4.10. Western Blot Analysis

The proteins were extracted with a radioimmunoprecipitation assay buffer (150 mM NaCl, 1% Triton X-100, 1% sodium deoxycholate, 0.1% SDS, 50 mM Tris-HCl, and 2 mM EDTA) supplemented with a protease and phosphatase inhibitor cocktail (Roche, Mannhem, Germany). Protein concentrations were determined using a BCA assay kit (Thermo Scientific, Chicago, IL, USA). Equal quantities of the protein were subjected to SDS-PAGE (10–12% gel) and transferred to polyvinylidene difluoride membranes (Millipore Corp., Billerica, MA, USA). After blocking in 5% skim milk, the membranes were incubated overnight at 4 °C with antibodies specific to phosphorylated p44/42 MAPK, ERK, phosphorylated GSK-3, GSK3β, phosphorylated Akt (Ser473), Akt, phosphorylated p38, p38, phosphorylated JNK, JNK, phosphorylated CREB, p53 and GAPDH; all primary antibodies were diluted 1:1000 in the blocking solution. Further, the membranes were incubated with the corresponding secondary antibodies and developed using a chemiluminescent reagent (Thermo Scientific). Relative intensities of the specific protein bands were quantified using the ImageJ software.

### 4.11. Statistical Analysis

Data are presented as mean ± S.D. from three or more independent experiments. Data analysis was performed using one-way ANOVA followed by Tukey’s post hoc multiple comparisons test (GraphPad software, Inc., La Jolla, CA, USA). A probability value of *p* ˂ 0.05 was considered statistically significant.

## 5. Conclusions

In this study, we demonstrated that sulfuretin prevents MPP^+^-induced apoptotic cell death in SH-SY5Y cells. It effectively decreased MPP^+^-induced ROS accumulation, p53 expression, and Bax/Bcl-2 ratio, and increased MPP^+^-induced reduction in MMP. Furthermore, it phosphorylated Akt/GSK3β and ERK signaling pathways dephosphorylated by MPP^+^. Taken together, sulfuretin protects against MPP^+^-induced cytotoxicity through the activation of Akt/GSK3β and ERK signaling pathways in SH-SY5Y cells. This study helps to understand the neuroprotective mechanism of sulfuretin in PD. Our in vitro data warrant further in vivo studies to substantiate the therapeutic effects of sulfuretin in PD. 

## Abbreviations

DCFH-DA	2′,7′-Dichlorodihydrofluorescein diacetate
PD	Parkinson’s disease
PARP	PolyADP-ribose polymerase
ROS	Reactive oxygen species
MMP	Mitochondrial membrane potential
MPP^+^	1-Methyl-4-phenyl pyridinium
MAPKs	Mitogen-activated protein kinases
ERK	Extracellular-signal-regulated kinase
6-OHDA	6-hydroxydopamine
